# Innovations in health information technologies for chronic pulmonary diseases

**DOI:** 10.1186/s12931-016-0354-3

**Published:** 2016-04-05

**Authors:** Blanca E. Himes, Elissa R. Weitzman

**Affiliations:** Department of Biostatistics and Epidemiologyok, University of Pennsylvania, Philadelphia, PA 19104 USA; Computational Health Informatics Program, Boston Children’s Hospital, Boston, MA 02115 USA; Division of Adolescent Medicine, Boston Children’s Hospital, Boston, MA 02115 USA

## Abstract

Asthma and chronic obstructive pulmonary disease (COPD) are common chronic obstructive lung disorders in the US that affect over 49 million people. There is no cure for asthma or COPD, but clinical guidelines exist for controlling symptoms that are successful in most patients that adhere to their treatment plan. Health information technologies (HITs) are revolutionizing healthcare by becoming mainstream tools to assist patients in self-monitoring and decision-making, and subsequently, driving a shift toward a care model increasingly centered on personal adoption and use of digital and web-based tools. While the number of chronic pulmonary disease HITs is rapidly increasing, most have not been validated as clinically effective tools for the management of disease. Online communities for asthma and COPD patients are becoming sources of empowerment and support, as well as facilitators of patient-centered research efforts. In addition to empowering patients and facilitating disease self-management, HITs offer promise to aid researchers in identifying chronic pulmonary disease endotypes and personalized treatments based on patient-specific profiles that integrate symptom occurrence and medication usage with environmental and genomic data.

## Background

Asthma and chronic obstructive pulmonary disease (COPD) are common chronic obstructive lung disorders. Asthma, an inflammatory disease characterized by variable airflow limitation and airway hyperresponsiveness, affects over 25 million Americans [1]. COPD, characterized by alveolar destruction, shortness of breath, cough, and sputum production, is estimated to affect over 24 million Americans [2]. Exacerbations, which are episodes of worsening symptoms requiring the use of systemic corticosteroids or other treatments to prevent serious outcomes, are a major cause of asthma and COPD morbidity and health care costs that in severe cases lead to death [3–6]. There is no cure for asthma or COPD, but clinical guidelines exist for controlling symptoms, preventing exacerbations and improving lung function in patients with these diseases [7, 8]. Thus, the goal of clinical therapy for most asthma and COPD patients is to prevent lung damage and control symptoms so that these do not interfere with daily activities.

The pathobiology of asthma and COPD are distinct although both diseases share some features. Asthma is often a childhood disease, but some patients continue to manifest symptoms or develop the disease as adults. The inflammatory changes that accompany asthma include increased T_H_2-type cells, increased eosinphils, mast cells, and lymphocytes in the airways, increased airway responsiveness to specific exposures or exercise, and in the case of severe and/or persistent disease, airway remodeling (i.e. increased airway smooth muscle mass, epithelial goblet cell hyperplasia) [9]. COPD is an adult disease whose leading cause is long-term cigarette smoking. The most traditionally recognized forms of COPD are chronic bronchitis, characterized by bouts of coughing and mucous production, and emphysema, characterized by damaged alveolar structure [10]. Adding to the complexity of managing asthma and COPD are the heterogeneity of symptoms and characteristics exhibited by patients. Both diseases are now recognized as being composed of several endotypes that have been, and continue to be, identified via precise phenotyping and association with molecular signatures [11–13].

Environmental exposures, including allergens, viruses, bacteria, and air pollutants, play a critical role in the development of asthma and COPD, as well as the triggering of disease exacerbations [14]. However, the response to environmental factors is complex and varies according to each individual’s genetic landscape and history of previous exposures, making it difficult to understand the precise mechanisms by which exposures lead to disease. Management of asthma and COPD includes limiting exposure to known triggers of exacerbation and seeking to identify unknown triggers. Some patients (and their parents, in the case of childhood asthma) keep track of environmental exposures in an effort to better understand and manage their disease, but currently, there are no efficient ways to quantitatively monitor personal exposures.

The most common pharmacologic treatment of asthma and COPD consists of inhaler-based bronchodilator and corticosteroid medications [10, 15]. Short acting bronchodilators (i.e., β_2_-agonists for asthma and COPD, anticholinergics for COPD) are “rescue” medications used to provide quick symptom relief [15]. Because increased use of bronchodilator medication correlates with worsening symptoms, keeping track of their use can serve as an indicator of exacerbations. Inhaled corticosteroids are anti-inflammatory “controller” medications that have been shown to improve clinical outcomes, including decreased symptoms and exacerbations, among asthma and COPD patients [8, 15]. Long acting bronchodilators, increasingly used in combination with inhaled corticosteroids, are commonly used for asthma and COPD patients with more severe disease [10, 15]. Not all asthma and COPD patients respond to currently available therapies, and the development of novel treatment approaches is ongoing. However, patients are not always adherent to their prescribed asthma and COPD medication plans, with non-adherence estimates ranging between 30 % and 70 % [16, 17]. Due to medication non-adherence, it can be difficult to distinguish patients with severe, medication insensitive disease from those who do not take medicines appropriately.

Health information technologies (HITs) are revolutionizing healthcare and becoming mainstream tools to assist patients in self-monitoring and decision-making, including in models whereby patients share data with providers [18–20]. Because there is a dearth of evidence that direct-to-consumer HIT tools are effective or that they provide accurate disease recommendations, such HITs are not widely used in clinical practice [21]. Nonetheless, the use of HITs is becoming widespread in healthcare, industry and research. For example, social media data is being used for the surveillance of infectious [22] and chronic diseases [23] on its own, and in conjunction with patient samples [24]. Successful models of online social networks for patient support coupled with participatory surveillance for health research exist, such as the TuDiabetes.org community for individuals with diabetes [25]. Large companies such as Apple and Google have also launched their own HITs. Specifically, Apple has created two software frameworks that facilitate the creation of healthcare apps: ResearchKit (released March 2015), designed to assist in medical research patient enrollment and data collection, and CareKit (released March 2016), designed for patient-centered disease self-management (http://www.apple.com/researchkit/). Although the use of these recently launched Apple products is limited, several promising academic partnerships focused on the study specific diseases have formed. Verily, a Google offshoot, represents another major effort focused on developing and using HITs to improve health (https://verily.com/). This company goes beyond apps, to also developing hardware and software to answer biomedical questions via sophisticated HITs. Verily partners with academic researchers, and notably, one of its partnerships with Vanderbilt University was selected to launch the first pilot of the Precision Medicine Initiative in February 2016. Beside HIT tools that are provided directly to patients, hospitals and healthcare systems are designing their own [18, 19]. The strong momentum behind development of direct-to-consumer and clinical HITs for chronic disease management are testimony to the widespread paradigm shift in healthcare toward a greater reliance on HIT-enabled approaches. Here, we describe the current landscape of HITs for chronic pulmonary diseases, and the opportunities they offer to improve patient outcomes, clinical care, and research.

### Domains where HITs offer promise for chronic pulmonary diseases

#### Disease self-management

Management of chronic pulmonary diseases includes decision-making based on patient symptoms, environmental exposures, and medication usage. Helping patients understand how these variables influence their health and, when necessary, instructing them on how to take medications properly and seek care, empowers them to develop skills that can change their health behavior and fulfill health related goals [26–28]. Validated symptom diaries for both children [29] and adults [30] that were developed for clinical trial and intervention studies, rather than to directly provide feedback to patients, can be applied to self-monitoring. Previous studies have found that asthma and COPD self-management programs that include symptom diaries and instructions on medication usage, are associated with better disease control, enhanced quality of life and fewer hospital visits [31, 32]. HITs, and in particular mobile health (mHealth) apps, are ideally suited to aid in chronic disease self-management [33].

Early asthma and COPD apps were created as either (1) general information tools to explain symptoms, the use inhaler medications, and present other educational material or (2) to replace paper-based and electronic patient diaries of symptoms, triggers, and pulmonary function measures (e.g., peak flow meter results). Previous reviews have concluded that apps offer promise, but have not been shown to be effective [21, 34–36]. A 2012 review of 103 asthma mobile apps found that 56 were solely sources of asthma information and 47 were asthma management apps that could be used to electronically record pulmonary function test (PFT) measures and symptoms [34]. The validity of many apps was questionable: 32 of 72 apps that provided recommendations gave ones that were not supported by clinical evidence, only 55 % of apps provided creator contact details, only 18 % stated a funding source, and only 17 % stated a confidentiality policy [34]. In 2015, an updated review of asthma apps by the same authors found that the number of apps between 2011 and 2013 had increased from 93 to 191, despite withdrawal of 25 % of the 2011 apps, while the general characteristics remained unchanged: 50 % of apps functioned as information sources only and 24 % served as simple electronic diaries [35].

A 2013 Cochrane review that focused on the feasibility and cost-effectiveness of asthma apps to improve symptom control screened hundreds of articles and subsequently focused on the only two that were randomized control studies involving use of an app that equipped users with one or more asthma self-management skills [36]. One of these studies that took place in Taiwan found that use of an app increased asthma control vis-à-vis increased peak flow rate, better quality of life, fewer exacerbations and increased adherence to inhaled corticosteroids [37]. The second study, which took place in the UK, found that use of an app to track daily medication usage and peak flow readings, compared to paper based monitoring, did not improve asthma control or increase self-efficacy based on differences in exacerbations, steroid courses or unscheduled doctor’s visits [38]. While educational and diary apps can be helpful, apps have the potential to do much more than provide general information or enable record keeping.

The ability to longitudinally collect symptom, trigger, and PFT data permits the detection of significant changes over time to help patients determine whether they are following their medication plan or are having worsening symptoms, but there are currently no effective, large scale methods for chronic pulmonary disease surveillance. Presentation of relationships among symptoms, triggers and PFTs using visually intuitive graphical reports to patients has the potential to reinforce positive behaviors (e.g. medication adherence) as well as provide alerts when symptoms might worsen (e.g. downward pulmonary function trend). Apps can provide fixed reminders based on patient medication plans according to patient preference, and compare actual medication usage to that of medication plans to help determine whether patients are indeed following their plans and/or suggest that their plans need to be changed. Data from external information sources, including weather, allergen, and air quality reports can be integrated with user-specific data to deliver notifications based on known patient triggers as well as to detect novel relationships among these variables and identify unknown triggers.

Three examples of mHealth apps that use technology in novel ways to enhance chronic pulmonary disease self-management are Wizdy (LifeGuard Games, Inc., Brighton, MA), Care TRx (Gecko Health Innovations, Inc., Cambridge, MA), and Propeller Health (Propeller Health, Madison, WI). The Wizdy Pets app is designed to help children with asthma understand their disease by learning about asthma triggers and the importance of taking medications. It accomplishes this via a game that children play involving taking care of a fire-breathing dragon “pet” that has asthma. Care TRx and Propeller Health apps work in conjunction with Bluetooth-enabled sensors that attach to standard inhaler medications and can wirelessly record time and location of medication usage (the latter when sensors are synced with a smartphone). The apps provide inhaler usage reports and asthma management guidance, incorporate external information about potential asthma triggers, and provide intuitive graphs to users. A pilot study of 30 individuals using Propeller Health sensors for four months found that asthma control increased as measured by Asthma Control Test (ACT) scores during months 2–4 of the study [39]. A subsequent randomized control trial of Propeller Health sensors in 495 patients (245 receiving routine care; 250 with receiving sensor-based feedback) found that those receiving feedback had decreased daily short-acting beta agonist (SABA) use during the study period (up to 1 year per person): 0.19 in routine care arm vs. 0.14 in intervention arm [40]. This effect was more pronounced among those participants who began the trial with uncontrolled asthma: mean daily SABA per person for the study period was 0.25 in routine care arm vs. 0.19 in intervention arm [40]. Although the effect sizes were relatively small, adults who initially had uncontrolled asthma and received feedback had improved ACT scores relative to those receiving usual care [40]. Although the Care TRx and Propeller Health apps have been designed for both asthma and COPD patients, published tests of their utility have focused on asthma management. Thus, while apps such as these offer promise to improve disease self-management, further studies in larger and diverse populations are needed to show that they are clinically effective [21].

#### Health care performance

Use of apps for disease self-management has the potential to improve health care performance when these are designed as telehealth interventions to enable shared decision-making and proactive care by both patients and healthcare providers—core elements of the chronic care model [41, 42]. Data from apps can be sent directly to healthcare providers where it can be incorporated into health records and used to actively care for patients. Clinical decision support systems can be built to aid providers in identifying individuals who are not adhering to recommended treatment, are at risk for exacerbations, and/or have uncontrolled disease. Alternatively, patients can use information gathered from such apps to seek care and share data with providers at the point-of-care. The end result of either system might be improved health care performance.

Poor medication adherence—skipping medicines or not taking them as prescribed—has been correlated with adverse clinical outcomes, and increased healthcare utilization and mortality [16, 17, 43]. In the case of asthma, current controller therapies (e.g., inhaled corticosteroids) decrease the risk for exacerbations and hospitalizations by 30-50 % [44], suggesting that asthma morbidity and costs would decrease significantly if the disease were better managed in a greater number of patients. Previous efforts to address undertreatment and medication non-adherence among asthma patients, directed at both patients [45–47] and providers [48], have had mixed success, but HITs offer novel opportunities for improved design. Inclusion of educational materials with HITs and/or in-person education prior to long-term use of HIT to monitor diseases is critical as inhaler technique training has been found to be essential for proper symptom control [49, 50]. By providing educational material and actionable feedback via apps and other HIT tools, population care of patients would be better supported (e.g., improved panel management gives access to timely and objective medication use data) and interventions would be more personalized (i.e., patient-tailored approaches would be informed by larger amounts of patient-specific data). This type of feedback would help shift chronic disease management from a reactive stance—where high-risk patients come to the attention of their healthcare providers *after a problem has occurred*—to a proactive stance where at-risk patients are identified early and supported to avoid an exacerbation or problem.

#### Patient communities

Web-based communities and social media platforms have helped to engage and support patients more successfully than has ever been possible. According to a 2010 estimate, 62 % of US adults living with at least one chronic disease go online [51]. While this percentage is lower than that for adults with no chronic diseases (81 %), it represents a significant percentage that will likely continue to increase. In addition to being online, many adults are users of social media: an estimated 71 % of online adults in the US are Facebook users, representing 58 % of all American adults [52]. PatientsLikeMe, an online patient community with over 400,000 members (http://www.patientslikeme.com) has been a successful resource for patients to connect with each other, share experiences, and contribute data for research on several diseases, including amyotrophic lateral sclerosis [53], epilepsy [54] and multiple sclerosis [55]. Owing to their large size and engaged community, PatientsLikeMe has become a leading driver and enabler of many patient-centered outcomes projects. While this organization has not focused on chronic pulmonary diseases, its membership does include 5,849 patients with asthma and 2,348 patients with COPD (based on search performed February 1, 2016).

Although not all are widely known, online communities focused on chronic respiratory diseases exist. The Asthma and Allergy Foundation of America (AAFA, Landover, MD), the largest asthma advocacy organization in the US, in partnership with Inspire (Princeton, NJ), an online patient engagement platform, offers a website (http://www.inspire.com/groups/asthma-and-allergy-foundation-of-america/) where patients and other stakeholders can connect and provide support to one another via online discussions and educational resources. On February 1, 2016, the site listed 3,532 members. Another online community focused on asthma is the AsthmaCommunityNetwork (http://www.asthmacommunitynetwork.org), which is sponsored by the US Environmental Protection Agency, the Allies Against Asthma program, and the Merck Childhood Asthma Network, Inc. nonprofit organization. The main goal of this site (3,619 members as of February 1, 2016) is to provide educational resources to improve asthma care, including the assignment of mentors to help with asthma management plans and discussion forums. The COPD Foundation (http://www.copdfoundation.org) created an integrated patient and physician registry called COPD360 and sponsored by AstraZeneca Pharmaceuticals, LP (Wilmington, DE). This effort, which aims to gather data for 125,000 patients to identify new COPD treatments, is the largest effort in COPD that includes patient-centered research.

Membership in online communities is free to individuals and advocacy groups, but most have business models in which profit from clinical trial recruitment, consumer health research, and advertising directed at its members offset the costs of hosting and maintaining the communities. This point raises concern with some, but most members of patient communities are happily engaged users who place trust in website creators to safeguard their privacy and advocate on behalf of patients with pharmaceutical and other industries. The existence and success of online communities relies on patients who are “information altruists” willing to engage with caregivers, investigators and other stakeholders to understand their diseases and seek cures [56, 57]. While there are no large-scale published reports based on data from online communities of chronic pulmonary disease patients, as these communities and patient-centered research efforts grow, their value in improving health outcomes may become better established.

#### Health disparities

Disparities in chronic pulmonary diseases are a well-known problem in the US, with persisting and marked differences by race/ethnicity and socioeconomic status [58, 59]. For example, asthma prevalence among black children is nearly twice that among white children (16.0 % vs. 8.2 %), while prevalence among Puerto Ricans is 2.6 times greater than among Mexican Americans and 1.7 times greater than among non-Hispanics (16.9 % vs. 6.5 % or 10.0 %) [60]. Less published studies on COPD disparities are available than for asthma, but low socioeconomic status has been associated with COPD severity and exacerbations [61]. Chronic pulmonary health disparities are typically attributed to poor health, disease risk factors and limited access to health care due to social, economic and environmental disadvantages [59]. Potentially modifiable factors that can contribute to these disparities are medication non-adherence and health literacy [16, 17, 27, 28].

The shift in healthcare toward a greater reliance on HIT-enabled approaches necessitates an understanding of the acceptability and utility of these approaches among *all* patients, including those who are disproportionately affected by diseases. In the case of chronic pulmonary diseases, most HIT tools have not been designed to address the concerns and barriers faced by racial/ethnic minority groups or those of low socioeconomic status who are disproportionately affected by asthma and/or COPD, leaving open the question as to whether the proliferation of HIT tools will narrow or widen existing disparities. Such tools must be designed and tested in diverse populations to ensure they will work for *all* including those from racial/ethnic groups who are at peak risk. One concern about the proliferation of mHealth apps in particular is that individuals of lowest socioeconomic groups will not be able to use such apps because they do not have access to smart phones. However, there is evidence that mHealth apps do have the ability to reach diverse groups and individuals in low income households: many communities of color use mobile technology to engage online in lieu of having a home internet connection [62]. According to Nielsen company estimates from 2010 in the US, 45 % of Asians and Pacific Islanders, 45 % of Latinos, and 33 % of African Americans owned smartphones compared to 27 % of Caucasians [63]. A 2013 Pew Research Center report, estimated that among cell phone users, 15 % of black and 11 % of Hispanic vs. 7 % of white users had a mobile phone app to help manage disease [64]. Thus, mHealth apps and online communities may actually serve to reduce chronic pulmonary diseases disparities, if they are found to be acceptable and effective for diverse groups.

#### Research studies

In addition to aiding in self-management and care of patients based on our current knowledge of chronic pulmonary diseases, HITs provide unprecedented opportunities to gain insights into these diseases via research studies. Online patient communities serve as a source of engaged patients who are interested in understanding their diseases and are willing to provide helpful insights regarding patient-centered outcomes and factors that are often overlooked in traditional care models. Participatory research studies for chronic pulmonary diseases based on these online communities are in their initial stages. Beyond facilitating subject recruitment, HITs make possible the collection and analysis of novel data types.

The use of sensors on inhaler medications that can conveniently track longitudinal geospatial data on use of controller (e.g., inhaled corticosteroid) and rescue (e.g., short-acting beta agonist) medications allows for the differentiation of patients with uncontrolled or severe disease vs. those who are non-adherent to their treatment regimens. Making this distinction in large numbers of individuals is often a limitation of traditional epidemiological studies that rely on journal entries or healthcare utilization data only to define exacerbations. Besides increasing individual-level resolution of phenotypes, tracking longitudinal geospatial data on rescue medication usage in a large number of subjects would be helpful to identify locations that are significantly associated with increased exacerbations, and subsequently, identify environmental variables (e.g., air quality information, pollen, ultraviolet sensitivity index levels, housing codes) that are also associated with such regions. Ideally, capturing individual-level time-dependent variation of all relevant environmental exposures would be possible. As sensors for environmental exposures become cheaper and more convenient, these more precise environmental measures will be linked to medication usage data, allowing for increasingly personalized patient profiles. Similarly, as obtaining genomic data for individuals becomes cheaper and more convenient, it is likely that transcription profiles from sputum or blood representing patients’ profiles as well as those of their pathogens, will be obtained as patients experience differences in symptoms. It may soon become possible to complement transcriptomic data with metabolomics markers from sputum and exhaled condensates. Integration of symptom, medication usage, environmental, and genomic data types would allow for a more comprehensive understanding of chronic pulmonary diseases than is currently possible, and thus, aid in more precise diagnoses, improved prediction of poor outcomes, and the creation of personalized therapies.

Two ResearchKit apps that attempt to capture symptom data for a large number of chronic pulmonary diseases patients are the Asthma Health app, co-developed with LifeMap, Mount Sinai, and Weill Cornell Medical College researchers, and the Stop COPD app, co-developed with DatStat as part of the COPD Foundation’s Patient-Powered Research Network. The study using the Asthma Health app, which currently has over 8,800 enrolled participants, aims to track individual symptoms and relate them to environmental measures, as well as to provide education, self-monitoring, and adherence feedback (http://apps.icahn.mssm.edu/asthma/). Further, genetic data from Asthma Health participants who are also 23andme (http://www.23andme.com/) customers is being integrated as of March 2016, enabling gene association studies that include a wealth of symptom and geospatial data.

## Conclusions

HITs are revolutionizing healthcare by becoming mainstream tools to assist patients in self-monitoring and decision-making, and subsequently, driving a shift toward a care model increasingly centered on *personal* adoption and use of digital and web-based tools. While the number of chronic pulmonary disease HITs is rapidly increasing, most have not been validated as clinically effective tools for the management of disease. For HITs to be effective, particularly for patients from socioeconomic and racial/ethnic groups that are disproportionately affected by chronic pulmonary diseases, these must be designed with input from *all* patients. Online communities for asthma and COPD patients are becoming sources of patient empowerment and support, as well facilitators of patient-centered research efforts. HITs will aid researchers in further identifying chronic pulmonary disease endotypes and personalized treatments based on patient-specific profiles that integrate symptoms and medication utilization with environmental and genomic data.

## Abbreviations

ACT: asthma control test
COPD: chronic obstructive pulmonary disease
HIT: health information technology
mHealth: mobile health
SABA: short-acting beta agonist
